# Obesity, Low-Grade Chronic Inflammation, and Clinical Outcomes in Spondyloarthritis: A Translational Synthesis

**DOI:** 10.3390/metabo16050347

**Published:** 2026-05-21

**Authors:** Andrej Belančić, Mislav Radić, Marija Rogoznica Pavlović, Marijana Vučković, Petra Šimac Prižmić, Elvira Meni Maria Gkrinia, Josipa Radić, Almir Fajkić

**Affiliations:** 1 Department of Basic and Clinical Pharmacology and Toxicology, Faculty of Medicine, University of Rijeka, Braće Branchetta 20, 51000 Rijeka, Croatia; 2 Division of Rheumatology, Allergology and Clinical Immunology, Department of Internal Medicine, Center of Excellence for Systemic Sclerosis in Croatia, University Hospital of Split, 21000 Split, Croatia; mislavradic@gmail.com (M.R.); psimacr@gmail.com (P.Š.P.); 3 Internal Medicine Department, School of Medicine, University of Split, 21000 Split, Croatia; josiparadic1973@gmail.com; 4 Hospital for Medical Rehabilitation of Heart and Lung Diseases and Rheumatism ‘Thalassotherapia-Opatija’, 51410 Opatija, Croatia; marija.rogoznica@gmail.com; 5 Division of Nephrology and Dialysis, Department of Internal Medicine, University Hospital of Split, 21000 Split, Croatia; maryanchi.1@gmail.com; 6 Independent Researcher, 11741 Athens, Greece; elvira.gkrinia20@alumni.imperial.ac.uk; 7 Department of Pathophysiology, Faculty of Medicine, University of Sarajevo, 71000 Sarajevo, Bosnia and Herzegovina; almir.fajkic@mf.unsa.ba

**Keywords:** chronic inflammation, obesity, spondyloarthritis

## Abstract

This translational synthesis highlights the potential role of obesity-induced low-grade chronic inflammation in modulating clinical outcomes among patients with spondyloarthritis (SpA). Obesity transforms adipose tissue into a pro-inflammatory endocrine organ, where hypertrophic adipocytes release adipokines such as leptin alongside cytokines including TNF-α and IL-6, potentially contributing to macrophage polarization toward an M1 phenotype and activating NF-κB signaling pathways. This systemic immunometabolic priming may lower activation thresholds at the enthesis—the primary pathological site in SpA—potentially amplifying IL-23/IL-17 axis activity via Th17 bias, innate-like lymphocyte responses, and stromal–immune crosstalk under mechanical stress. Clinically, patients with SpA and obesity have been reported to demonstrate heightened disease activity (BASDAI, ASDAS), impaired function (BASFI), accelerated radiographic progression (syndesmophytes, enthesophytes), and diminished biologic response rates, potentially attributable to pharmacokinetic alterations (e.g., subtherapeutic TNF inhibitor levels) and pharmacodynamic resistance. Multisystem comorbidities, including non-alcoholic fatty liver disease, cardiovascular events, metabolic syndrome, sleep disturbances, and depression, further exacerbate morbidity and diminish quality of life. Therapeutic implications emphasize obesity as a modifiable disease modifier. Weight loss interventions, including hypocaloric diets, anti-inflammatory regimens (e.g., Mediterranean diet), multicomponent exercise, GLP-1 receptor agonists, and bariatric surgery, have been associated with reductions in inflammatory biomarkers, improved remission rates (MDA, DAPSA), and prolonged drug survival by restoring adipokine balance and disrupting mechano-inflammatory loops. Future randomized controlled trials should prioritize long-term evaluations of integrated multidisciplinary strategies that combine metabolic optimization with immunomodulatory therapies, addressing adherence challenges through psychological support and patient-tailored protocols, while elucidating dose–response relationships for GLP-1RAs and exercise in diverse SpA subtypes to establish precision management paradigms that mitigate cardiometabolic burden and improve holistic outcomes.

## 1. Introduction

Spondyloarthritis (SpA) comprises a heterogeneous group of diseases that can affect the spine, sacroiliac joints, entheses, ligaments, and peripheral joints [1]. Based on predominant symptoms, SpA is divided into two major groups, axial SpA (axSpA) and peripheral SpA (pSpA), frequently with related disorders [2]. AxSpA primarily affects the spine and hips, causing inflammatory back pain and stiffness. Ankylosing spondylitis (AS) is the classic form. In contrast, pSpA usually involves peripheral joints, tendons, and entheses. Psoriatic arthritis (PsA) is a typical representative. The pathophysiology of SpA is not fully understood and involves complicated interactions between genetic predisposition and environmental factors that trigger immune system activation [3].

Obesity has been recognized as a contributing factor in the development of SpA [4] and is associated with an increased risk of rheumatic diseases, including rheumatoid arthritis (RA) and PsA, with reported odds ratios ranging from 1.2 to 6.56 [5]. Notably, epidemiologic data suggest that obesity promotes the development and progression of psoriasis via pro-inflammatory mechanisms and increases the risk of PsA [4,6]. In particular, the elevated incidence of cardiovascular risk factors and the resulting cardiovascular morbidity are shared with metabolic syndrome (MetS) [7]. Obesity may impair treatment response through both pharmacokinetic and pharmacodynamic mechanisms. Pharmacokinetically, increased adipose tissue leads to a larger volume of distribution and altered drug exposure, particularly for fixed-dose biologics such as TNF inhibitors, potentially resulting in subtherapeutic drug levels and reduced clinical efficacy (“dose–exposure mismatch”). Pharmacodynamically, obesity represents a chronic low-grade inflammatory state characterized by elevated TNF-α, IL-6, and adipokines such as leptin, which promote Th17 polarization and sustain IL-23/IL-17 axis activity. This persistent inflammatory milieu may attenuate the effectiveness of cytokine-targeted therapies and contribute to heterogeneous or incomplete clinical responses.

In addition, adipose-driven systemic inflammation may reinforce parallel inflammatory pathways (TNF and IL-23/IL-17), while mechanical stress at the entheses may further amplify local inflammatory signaling, collectively limiting the degree of clinical improvement despite treatment. Importantly, successful treatment of obesity may improve clinical outcomes in SpA by reducing systemic inflammatory tone, restoring adipokine balance, and improving pharmacokinetic drug exposure, thereby enhancing response to biologic therapy and facilitating disease control.

Further, adipose tissue-derived mediators, including adipokines dysregulated in obesity and MetS, have been proposed as mechanistic links between psoriatic disease activity and cardiovascular comorbidity [8,9]. According to the World Health Organization (WHO), overweight and obesity reflect abnormal or excessive fat accumulation that may impair health, and body mass index (BMI) remains a commonly used population measure [10].

Overweight and obesity are becoming a global issue and are linked to higher mortality and morbidity, cardiovascular and metabolic disorders, and some types of cancer [11]. The prevalence of obesity among patients with axSpA is 13.5% and 27.4% in those with PsA [12,13].

Increased BMI has been associated with worse physical function, higher symptom burden, and reduced treatment response in patients with SpA [14,15]. Patients living with obesity have higher subjective disease activity and fewer benefits of exercise [12]. Long-term biomechanical stress over the joints and entheses brought on by obesity has been shown to be a significant risk factor for the development of SpA in animal models [16].

**Figure 1:**
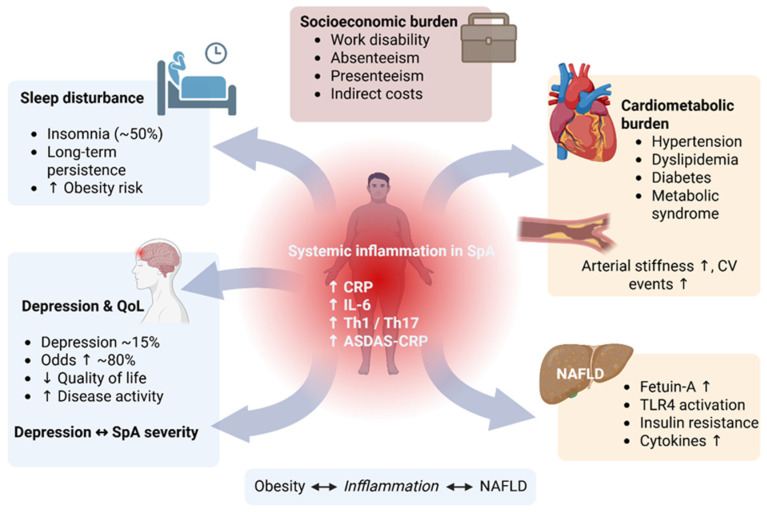
Cardiometabolic and multisystem burden of spondyloarthritis driven by obesity. Abbreviations: SpA—spondyloarthritis; CRP—C-reactive protein; IL-6—interleukin-6; Th1/Th17—T helper 1/T helper 17 lymphocytes; NAFLD—non-alcoholic fatty liver disease; TLR4—Toll-like receptor 4; QoL—quality of life; CV—cardiovascular; ASDAS-CRP—Ankylosing Spondylitis Disease Activity Score based on C-reactive protein.

By integrating clinical evidence with molecular insights, this review aims to synthesize current knowledge on the role of obesity and low-grade chronic inflammation in SpA and their impact on clinical outcomes (Figure 1). Obesity is considered here primarily as a disease-modifying factor that reshapes clinical phenotype and treatment response, rather than as an independent trigger. This review first outlines the clinical and pathophysiological features of SpA, and then examines how obesity modifies these processes through increased systemic inflammation and metabolic dysregulation.

## 2. Narrative Review Methods

This study was conducted as a narrative review with scoping elements to integrate clinical evidence with molecular insights on the role of obesity and low-grade chronic inflammation in SpA. A targeted literature search was performed in PubMed and Scopus, supplemented by manual screening of reference lists of relevant articles. The search covered publications from 2010 onwards. Search terms included: obesity, overweight, adipose tissue, adipokines, immunometabolism, low-grade inflammation, SpA, ankylosing spondylitis, psoriatic arthritis, enthesitis, TNF-alpha, IL-6, IL-17, IL-23, Th17, NF-kB, and biologic therapy. Studies were included if they reported data on obesity-related inflammation, immunometabolic mechanisms, adipose tissue-driven cytokine signaling, or their association with SpA pathophysiology, clinical outcomes, or treatment response. Both experimental and clinical studies, as well as relevant high-quality reviews, were considered when contributing to mechanistic understanding or clinical translation of obesity–SpA interactions. Exclusion criteria included non-English publications, conference abstracts without full text, and studies not directly related to obesity-associated inflammation or SpA mechanisms or outcomes. Manuscript selection was further refined through expert consensus among the authors based on relevance, methodological quality, and contribution to the conceptual framework. Evidence was synthesized narratively and organized thematically to integrate mechanistic and clinical perspectives. As a narrative review, this work does not adhere to formal systematic review methodologies (e.g., PRISMA) and does not include structured risk-of-bias assessment; therefore, potential selection bias and heterogeneity of included studies should be acknowledged. Nevertheless, this approach is appropriate for synthesizing diverse evidence and providing a comprehensive, hypothesis-driven overview of complex and heterogeneous translational evidence.

## 3. Obesity as a Systemic Inflammatory State

Obesity is widely recognized not simply as excess adiposity, but as a state associated with chronic low-grade inflammation characterized by persistent immune system activation and systemic release of inflammatory mediators [17,18]. Central to this pathological inflammatory milieu is adipose tissue, which functions beyond a passive energy reservoir to act as a dynamic endocrine–immune organ. In obesity, hypertrophic adipocytes secrete a diverse array of chemokines and cytokines that recruit and activate immune cells, transforming the adipose microenvironment into a hub of pro-inflammatory activity. This state fosters an imbalance in adipokine production, favoring pro-inflammatory molecules such as leptin and monocyte chemotactic protein-1 overprotective adiponectin, thereby disrupting tissue homeostasis and promoting systemic immune dysregulation [18,19].

At a cellular level, lean adipose tissue is predominantly populated by regulatory immune cells including eosinophils and type 2 innate lymphoid cells that sustain homeostasis through secretion of type 2 cytokines such as interleukin-4, -5, and -13. These cytokines maintain macrophages in an anti-inflammatory, M2-like phenotype. Such lean adipose tissue macrophages produce and release anti-inflammatory cytokines (e.g., IL-1 receptor antagonist, IL-4, IL-10, TGF-beta1), and express arginase-1, which then blocks the activity of inducible nitric oxide synthase [18,19]. However, with notable weight gain leading to overweight and obesity, visceral adipose tissue undergoes low-grade chronic inflammation characterized by hypertrophy and hyperplasia of adipose tissue macrophages alongside a loss of tissue homeostasis. Concurrently, a type 1 interferon-gamma-driven inflammatory response arises, accompanied by a polarization shift in adipose tissue macrophages from an anti-inflammatory M2-like state to a pro-inflammatory M1-like phenotype. These M1 macrophages release substantial levels of pro-inflammatory cytokines including interleukin-1 beta, interleukin-6, interleukin-12, tumor necrosis factor-alpha, and monocyte chemotactic protein-1, which induce the production of inducible nitric oxide synthase [18,19,20,21].

Chronic inflammation in obesity is driven by dysregulated activation of key inflammatory signaling pathways, notably NF-kB and c-Jun N-terminal kinase, which together orchestrate systemic insulin resistance through complex and interconnected molecular mechanisms. These pathways are activated in metabolic tissues in response to obesity-associated stressors, including elevated levels of free fatty acids, adipocyte hypoxia, and the persistent secretion of pro-inflammatory cytokines. Upon activation, NF-kB and c-Jun N-terminal kinase disrupt insulin signaling cascades and establish a self-perpetuating inflammatory milieu that exacerbates metabolic dysfunction [18,22,23,24,25].

Importantly, the impact of obesity-related chronic inflammation extends far beyond adipose tissue, influencing multiple organ systems and driving widespread metabolic dysregulation. Circulating inflammatory mediators such as interleukin-6 and tumor necrosis factor-alpha interfere with hepatic metabolism by promoting gluconeogenesis, reducing glycogen synthesis, and stimulating acute phase protein production. Concurrently, these inflammatory signals impair skeletal muscle glucose uptake and mitochondrial oxidative capacity, contributing to metabolic inflexibility [18,22,24,26,27]. Furthermore, inflammatory cytokines exacerbate endothelial dysfunction and promote early atherogenesis through upregulation of adhesion molecules. Additionally, adipose-derived angiotensinogen activates the renin–angiotensin–aldosterone system, fostering vascular remodeling and hypertension [18,23,26]. Collectively, these systemic effects highlight the far-reaching consequences of obesity-induced inflammation on metabolic health.

Given these multifaceted mechanisms, obesity-related low-grade chronic inflammation represents a potential biological substrate linking excess adiposity to its spectrum of complications. Targeting adipose tissue dysfunction, immune activation, and inflammatory signaling pathways may help interrupt this pathogenic cascade. Comprehensive management strategies that integrate lifestyle modification, pharmacotherapy, or even invasive procedures aim to restore immunometabolic balance, reduce systemic inflammation, and ultimately improve clinical outcomes [18]. Notably, these inflammatory processes are highly relevant to musculoskeletal health, as chronic low-grade inflammation can exacerbate immune-mediated joint and entheseal pathologies. Particularly, SpA exemplifies a disease state where obesity-related inflammation may worsen disease activity, symptom burden, and treatment response, underscoring the importance of addressing systemic inflammation in this patient population.

## 4. Immunometabolic Links to Spondyloarthritis Pathophysiology

The following section summarizes key mechanisms linking obesity to SpA, focusing on pathways most relevant to clinical outcomes. SpA is often associated with HLA genetics and cytokines, as well as obesity and adiposity. A translational reading points to a shared entry mechanism: both behave as disorders of tissue-level barrier failure [28]. In SpA, the barrier is the enthesis and adjacent osteotendinous unit, which must resolve mechanical microdamage without converting repair into chronic inflammation. In genetically susceptible hosts, particularly those enriched for HLA-B27, microdamage is misread as persistent danger, innate sensing dominates, stromal cells shift from scaffolding to inflammatory drivers, and the TNF-alpha and IL-23/IL-17 axes become sustained rather than self-limited, a pattern distinct from synovial-first disease [29,30,31].

This barrier logic extends to the gut. Microscopic intestinal inflammation and dysbiosis can permit translocation of microbial products that amplify entheseal inflammation through the same TNF-alpha and IL-23/IL-17 pathways, reinforcing the enthesis as an active immunological interface rather than a passive mechanical site [28,29,31].

Although the mechanistic analogy is strong, obesity is not typically framed as a barrier failure in SpA research. Adipose expansion can outpace vascular adaptation, causing hypoxia, cell stress or death, and innate immune activation, while stromal–immune crosstalk sustains low-grade chronic inflammation. Therefore, obesity may theoretically produce a systemic inflammatory tone that reduces the threshold for entheseal inflammation and hinders resolution via the same cytokine circuits that are essential for SpA [29,30].

### 4.1. Low-Grade Metabolic Inflammation as a Primer of SpA Pathways

Because adipose tissue constantly exports metabolic and inflammatory signals, obesity is generally considered a systemic inflammatory state. As outlined in Section 3, hypertrophied adipose tissue exhibits immune remodeling and elevated TNF-alpha, IL-6, and leptin production, which may reduce activation thresholds across tissue-resident immune and stromal compartments [32,33].

Th17-leaning immunity is where this overlap is most noticeable. Leptin further promotes Th17 polarization, and obesity-associated immune remodeling can favor Th17 differentiation and IL-17 production. Increased IL-17/IL-23 signals have been observed in obesity, supporting the plausibility of IL-23/IL-17 tuning [34,35]. Obesity may function as a disease modifier that lowers the threshold for enthesis-predominant inflammation and can increase severity in predisposed hosts; readiness, not causality, is the bridge [36,37].

### 4.2. The Enthesis as an Immunometabolically Responsive Tissue

The enthesis is more than just a point of insertion. Tendon, ligament, fibrocartilage, bone, and local stromal and immune cells form a mechanically stressed interface in this specialized organ system that detects strain and microdamage and converts them into inflammation or repair [2]. Mechanosensitive stromal cells can trigger local inflammatory cascades when load surpasses repair capacity. This program is pathologically amplified in SpA, causing enthesitis and the formation of new bone, with stromal cells serving as important effectors [38].

Through biomechanical and biochemical processes, obesity may reinforce this axis. In addition to adding load, a metabolically inflammatory environment also releases TNF-alpha, IL-6, leptin, free fatty acids, and other mediators that intensify pro-osteogenic remodeling, oxidative stress, and NF-kB signaling. As a result, mechanical microtrauma and systemic metabolic inflammation converge at the enthesis [30,38].

Crucially, the enthesis contains IL-23-responsive, IL-17-producing tissue-resident and innate-like immune subsets, supporting initiation and persistence of local inflammation without classical antigen-driven autoimmunity, consistent with SpA as dysregulated tissue homeostasis and repair [39].

### 4.3. IL-23-Responsive Innate-like Lymphocytes: Why Metabolic Context Matters

A distinctive feature of SpA is the prominence of IL-23-responsive, innate-like T cell populations capable of producing IL-17A and IL-22, a central effector module emphasized in SpA immunobiology [40]. Human and translational data support the role of IL-23 at the enthesis, with IL-17 and IL-22 shaping both inflammation and remodeling [31].

Obesity may not only increase inflammatory burden, but also enhance permissiveness. The metabolically inflammatory environment (TNF-alpha, IL-6, leptin, free fatty acids) supports increased IL-17 production and Th17 and Th17-derived programs, including polyfunctional IL-17-producing CD4 or CD8 populations linked to PsA [34]. Simultaneously, metabolic stress may increase NF-κB and oxidative stress signaling, enhancing cellular responsiveness to local danger signals and IL-23 stimulation [41].

In addition, emerging evidence suggests that lipid rafts, including cholesterol- and sphingolipid-rich microdomains within the plasma membrane, may represent an additional regulatory layer in immunometabolic signaling. These membrane platforms facilitate the spatial organization of receptors and downstream signaling molecules, including Toll-like receptors, T cell receptors, and cytokine receptors, thereby modulating signal transduction efficiency [42]. In the context of obesity, alterations in membrane lipid composition and cholesterol content may influence lipid raft structure and function, potentially enhancing pro-inflammatory signaling pathways such as NF-κB activation and cytokine production. This mechanism may further contribute to heightened immune responsiveness and sustained low-grade inflammation, although its specific role in SpA pathophysiology remains to be fully elucidated [43].

In metabolically primed tissue, the same local IL-23 pulse can cause a stronger IL-17A or IL-22 effector burst. This is significant at the enthesis, where IL-17 stimulates neutrophil-driven cascades and IL-22 connects to stromal and bone responses [41]. This clarifies the SpA paradox of inflammation and new bone formation: immune activation and stromal remodeling are coupled by IL-23/IL-17 signaling, and obesity may further skew this coupling in favor of maladaptive osteoproliferation [16].

### 4.4. TNF-Alpha-Driven Processes: Not Redundant, but Cooperative with IL-17/IL-23

TNF-alpha is not a parallel pathway in SpA. It appears to work with IL-17 at the tissue level to promote stromal cell outputs, leukocyte recruitment, and endothelial activation, consistent with reviews that describe IL-17 as a central node integrated into larger inflammatory networks [44]. TNF-alpha can prime stromal and endothelial cells for amplified IL-17 responses, increasing chemokines such as IL-8 and reinforcing neutrophil recruitment and feed-forward inflammation [45].

Two potential TNF-alpha-related amplifiers may be associated with obesity. Adipose macrophage activation strengthens systemic innate tone by increasing circulating TNF-alpha and associated mediators [32]. In metabolic inflammation, TNF-alpha can act in concert with saturated fats (e.g., palmitate) to increase IL-8 production through TLR4-dependent TRIF or IRF3 signaling, sharpening leukocyte trafficking cues [46]. Second, at mechanically stressed interfaces, metabolic stress may increase tissue responsiveness to TNF-alpha signaling [45].

Translationally, TNF-alpha blockade may remove a key driver, but residual IL-17/IL-23 permissiveness plus ongoing mechanical strain can sustain disease in some patients with obesity, explaining response heterogeneity without implying drug-class superiority [32,44,45].

### 4.5. Adipokines as “Metabolic Cytokines” Shaping Entheseal Immunity and Stromal Tone

Adipokines may provide a direct biochemical bridge between obesity and SpA. Leptin, adiponectin, visfatin, and resistin can modulate innate and adaptive immunity, cytokine release, and stromal remodeling, and associations with inflammatory and structural outcomes have been reported in axSpA [46,47].

Mechanistically, leptin is typically pro-inflammatory, promoting Th1 or Th17 polarization and TNF-alpha or IL-17-linked outputs, with links to higher activity and progression signals in some cohorts [48]. Although adiponectin is isoform-specific and context-dependent, it frequently counter-regulates TNF-alpha or IL-6 programs and may exhibit inverse relationships with disease burden [48]. Additionally elevated in obesity, visfatin and resistin can support TNF-alpha or IL-6 (and occasionally IL-17-related) signaling, potentially escalating entheseal inflammation [49].

Abdominal obesity is especially relevant because visceral adiposity favors a more pro-inflammatory cytokine or adipokine profile, and cytokine differences associated with abdominal obesity have been described in SpA [50,51]. Table 1 summarizes the inflammatory mediators most consistently reported in SpA patients with obesity compared with SpA patients without obesity. Overall, obesity appears to amplify the inflammatory profile of SpA rather than introduce a completely separate cytokine signature. The most consistent changes involve higher activity of IL-6, TNF-alpha, MCP-1, IL-1beta, IL-8, and the IL-23/IL-17 axis, while adipokine-related mediators such as leptin, resistin, visfatin, and reduced adiponectin may further support Th17 polarization, macrophage activation, stromal cell responsiveness, and entheseal inflammation. However, direct quantitative comparison across studies remains limited because published reports differ in SpA subtype, obesity definition, biological sample, assay platform, and reporting units. Therefore, the table should be interpreted as a structured synthesis of reported inflammatory mediator patterns rather than as a pooled quantitative meta-analysis. Sex-, gender-, and age-related differences between patients with SpA with and without obesity have been explored only to a limited extent. Available evidence suggests possible sex-related differences in biologic drug retention, no studies specifically addressing gender-related effects, and a tendency for patients with obesity to be older and to have more comorbidities [12].

### 4.6. A Unified Mechanistic Loop: Mechanical Strain, Metabolic Priming, and Stromal Effector Programs

**Figure 2:**
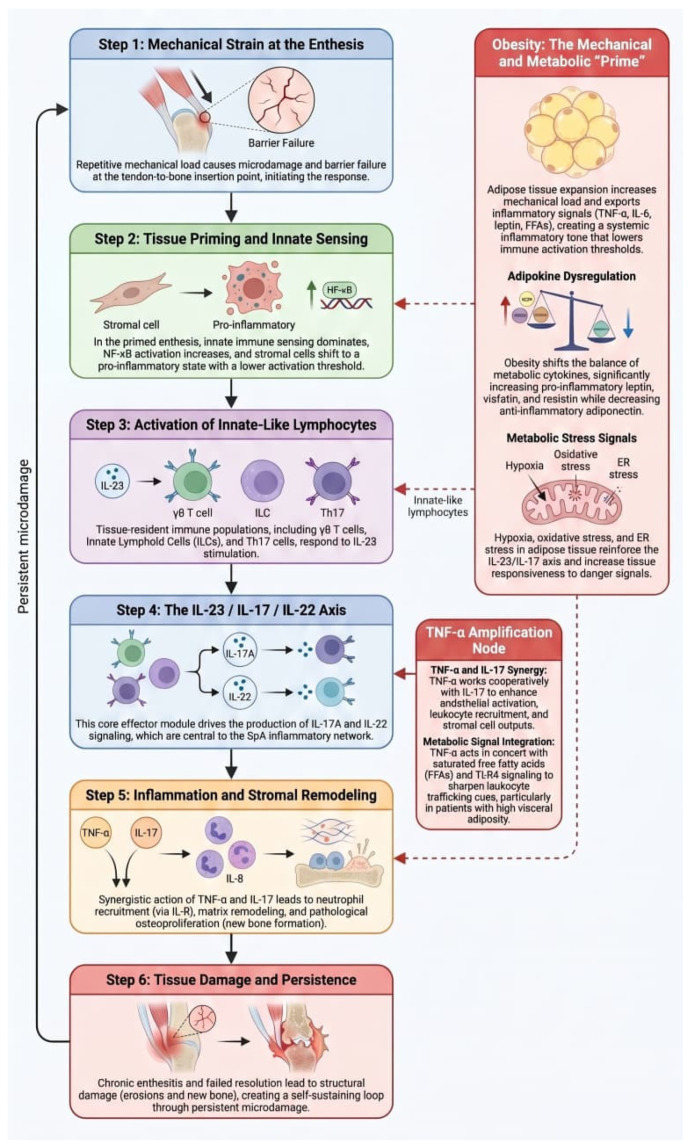
The mechanoinflammatory loop in spondyloarthritis: Obesity as a metabolic and mechanical amplifier. Abbreviations: SpA—spondyloarthritis; TNF-α—tumor necrosis factor alpha; IL—interleukin; IL-R—interleukin receptor; IL-17A—interleukin 17A; IL-22—interleukin 22; IL-23—interleukin 23; IL-8—interleukin 8; ILCs—innate lymphoid cells; Th17—T helper 17 cells; NF-κB—nuclear factor kappa B; FFAs—free fatty acids; ER—endoplasmic reticulum; TLR-4—Toll-like receptor 4.

A unified translational loop can be summarized as follows: tissue-resident immune subsets (such as IL-23R-expressing and innate-like populations) detect local danger signals produced by mechanical strain and repetitive microdamage at the enthesis. These subsets react to IL-23 cues by generating IL-17-family mediators and increasing local inflammation [52]. This program is executed by stromal cells at the enthesis through integration of mechanical and cytokine signals (e.g., IL-17, TNF-alpha). IL-22 is increasingly implicated as a link between immune activation and remodeling outputs [29]. Obesity may strengthen this loop by adding both more load (greater microdamage) and more priming (adipokines, elevated TNF-alpha or IL-6 tone, lipid mediators), lowering activation thresholds and slowing resolution, potentially contributing to persistence [53]. Because obesity amplifies the mechanoinflammatory circuit and adds to clinical heterogeneity, this framing explains why enthesitis is central to SpA and why obesity exacerbates phenotype without being the primary cause [29,53]. Figure 2 shows the mechanoinflammatory loop in SpA.

In addition to immunometabolic and mechanical factors, emerging evidence suggests that epigenetic mechanisms may represent an important interface linking obesity and SpA pathophysiology. Epigenetic modifications, including DNA methylation, histone modifications, and non-coding RNA activity, regulate gene expression without altering the underlying DNA sequence and may mediate interactions between environmental exposures and genetic susceptibility. In obesity, chronic metabolic stress and systemic inflammation have been associated with epigenetic reprogramming of immune and stromal cells, potentially influencing the expression of key inflammatory mediators such as TNF-α, IL-6, and IL-17 [54,55,56].

These epigenetic changes may affect immune cell differentiation and function, including macrophage polarization and Th17 responses, thereby contributing to persistent inflammation and altered tissue repair in SpA. In addition, variability in epigenetic profiles may partly explain heterogeneity in disease severity, clinical phenotype, and therapeutic response among patients. While the specific role of epigenetic regulation in SpA remains incompletely defined, this layer of regulation may provide further insight into disease mechanisms and support the development of personalized therapeutic approaches [54,55,56].

In summary, obesity intersects with SpA at the level of shared machinery: innate sensing, tissue-resident lymphocyte responsiveness to IL-23, cooperative TNF-alpha and IL-17 amplification, and stromal cell execution of both inflammation and remodeling. The enthesis sits at the center of this intersection as an immunometabolic sensor, translating metabolic stress and mechanical microtrauma into the clinical and structural signatures that define SpA. In addition, emerging evidence suggests that epigenetic regulation may further modulate these processes by influencing gene expression, immune cell function, and inflammatory pathway activity.

## 5. Influence of Obesity on the Clinical Phenotype of SpA

The processes by which obesity or overweight may affect axSpA are numerous. Initially, adipose tissue generates several inflammatory mediators (adipokines), hence initiating or exacerbating a pro-inflammatory condition in patients with axSpA [57]. Furthermore, biomechanical factors including aberrant loading, diminished trunk and lower extremity muscle mass, and dysregulated blood supply may exacerbate joint discomfort [57]. In people living with obesity, rapid atherosclerosis of the abdominal aorta and lumbar arteries can be noted, leading to disrupted perfusion of lumbar tissues, which may result in structural degradation and low back discomfort [57].

Maas et al. reported that patients with obesity were older, exhibited a higher prevalence of comorbidities, particularly arterial hypertension, and were less frequently HLA-B27 positive [12]. These data suggest a correlation between obesity and axSpA in the absence of HLA-B27. Given the cross-sectional design of the study, causal inferences cannot be made. Consistently, a markedly reduced prevalence of HLA-B27 positivity among patients with obesity was also observed in a prior retrospective analysis involving 155 patients with AS [58].

In a meta-analysis by Ortolan et al. [59], patients with axSpA and overweight or obesity had higher disease activity scores than patients with normal BMI; however, this difference was clinically meaningful mainly in patients with obesity and was more pronounced when disease activity was assessed by BASDAI rather than ASDAS. These findings align with prior studies indicating a significant correlation between elevated BMI and increased disease activity ratings [59,60]. Patients with axSpA and obesity exhibited elevated biomarkers (CRP and ESR) and demonstrated inferior physical function and QoL, as evaluated using questionnaires (BASFI and ASQoL), compared to overweight and normal weight patients [12]. In a short cross-sectional investigation, patients with AS and obesity exhibited elevated subjective markers of disease activity (BASDAI and VAS) and diminished physical function (BASFI) [60].

The justification for the correlation between obesity and PsA has been previously examined [5,41,61]. Obesity is characterized as a low-grade inflammatory condition, and both obesity and PsA share pathogenic inflammatory pathways [41,61]. Additional processes not directly linked to immune activation may also contribute to adverse outcomes in PsA. Obesity may correlate with biomechanical irregularities in the joints, especially in the lower extremities, potentially leading to heightened microdamage in these areas [14]. McGonagle et al. postulated that microscopic damage and the corresponding abnormal inflammatory response in the entheses may represent the earliest lesion in the progression of PsA [62]. The inclination of PsA towards the lower extremities may support this idea.

Another process may relate to altered pain thresholds. Obesity has been linked to increased levels of generalized pain in both healthy individuals and patients with RA [63,64]. Obesity is linked to osteoarthritis and many non-musculoskeletal comorbidities that might result in physical handicap independent of inflammatory arthritis [65]. HAQ scores are significantly affected by comorbidities in addition to the impact of arthritis [66].

Eder et al. demonstrated a correlation between elevated BMI and active disease across multiple domains, including pain, function (as measured by HAQ), painful joint count, patient global rating, and skin activity [14]. Shared inflammatory pathways may represent biological mechanisms linking obesity with psoriatic disease [41,48].

The observational cohort study by Vallejo-Yagüe et al. revealed that patients with obesity exhibited a substantial 49–58% decrease in the likelihood of attaining MDA, DAPSA remission, cDAPSA remission, and DAS28 remission within the initial year, in comparison to patients of normal weight [67]. In contrast, being overweight was solely linked to reduced likelihood of attaining DAPSA remission. In the groups with overweight and obesity, the correlation with attainment of DAPSA remission or low disease activity within the initial year and with 1-year treatment adherence was not statistically significant. Most individuals who attained MDA also obtained cDAPSA remission [67].

In the prospective study conducted by Di Minno et al., obesity was linked to a heightened chance of failing to attain MDA throughout a 12-month follow-up, in contrast to overweight [68]. Di Minno et al. also observed that elevated BMI forecasted a less favorable response to TNF-alpha inhibitors (TNFis) in individuals with PsA throughout a 24-month follow-up period [68]. The same group demonstrated that weight loss correlated with enhanced response to treatment with TNFi.

The principal discovery in the meta-analysis by Bakircic et al. was that elevated BMI was significantly associated with new bone growth, including syndesmophytes, enthesophytes, and a more advanced variant of the modified Stoke Ankylosing Spinal Spondylitis Score [15]. Limited research has examined the impact of BMI on peripheral enthesis, revealing a modestly significant connection between the Madrid Sonographic Enthesitis Index for ultrasound-assessed enthesitis and BMI [15]. The impact of BMI on sacroiliac joint and spinal inflammation remains ambiguous due to a lack of MRI-based investigations.

Physical and functional limitations associated with obesity or elevated disease activity might lead to physical inactivity, subsequently resulting in weight increase [12]. Physical activity is essential in managing SpA to preserve physical function and alleviate symptoms, and it is also advised to prevent obesity [69]. In addition to pharmacological interventions aimed at alleviating symptoms associated with disease activity, such as nonsteroidal anti-inflammatory drugs and/or TNFi, physical exercise appears crucial for disrupting the detrimental cycle of disease activity, physical inactivity, and obesity [12].

## 6. Pharmacologic Implications: Treatment Response and Drug Survival

There is increasing awareness of the impact of obesity on drug treatment response and survival in patients with SpA, an effect that extends beyond the well-established mechanical stress on joints [70]. Obesity constitutes a chronic, low-grade inflammatory condition that can influence drug response and bioavailability through adipose-derived mediators and inflammatory amplification, with downstream effects on cytokine networks relevant to SpA, including TNF-alpha, IL-6, and IL-17 [71]. As a result, adipose tissue interactions with TNF-alpha, IL-6, and IL-17 may reduce responses to cytokine inhibition and facilitate persistent systemic inflammation, worsening disease activity and altering the way biologic therapies are distributed, metabolized, and perceived by the immune system [71].

Based on the current results, obesity is associated with altered drug disposition due to increased distribution volume, modifications of absorption, and enhanced clearance, which may lower drug concentrations at therapeutic sites [72]. These effects are particularly evident in commonly used TNFis (adalimumab, etanercept, and certolizumab pegol), which are administered in fixed doses regardless of body weight [70]. In contrast, drugs whose dose is dependent on body weight, such as infliximab, may show less variability, although they can still be affected by changes in protein catabolism and vascular permeability driven by inflammation [70,73]. Patients with an elevated BMI may experience subtherapeutic drug concentrations, often referred to as a “dose–exposure mismatch”, contributing to reduced treatment efficacy [70].

In addition to pharmacokinetic effects, obesity may contribute to pharmacodynamic resistance through inflammatory amplification. Leptin, as an endocrine hormone, may enhance Th17 cell development and reduce Treg activity, thereby amplifying IL-17/IL-23-mediated inflammation [74]. This mechanism has been proposed as one explanation for the reduced efficacy of the TNF-alpha blockade and the high disease activity observed in patients with obesity, and may contribute to a “vicious cycle” in which increased inflammation accelerates drug clearance while reduced systemic exposure limits effective disease control [75].

Clinical evidence supports the above mechanisms. Real-world studies have reported reduced drug response and persistence in patients with SpA and obesity undergoing biologic therapy [76,77]. Particularly, patients with obesity receiving TNFis have a lower chance of achieving remission or low disease activity compared to normal-weight individuals [78,79,80]. The results for IL-17 and anti-IL-23 treatment are somewhat inconsistent. Although some studies have suggested that those drugs are not affected by body weight, their efficacy is influenced by other factors (SpA subtype, gender, prior exposure to biologics), and therefore definitive conclusions cannot be drawn [70,81]. Observational cohort data have also shown that women living with obesity may have greater retention of secukinumab, likely reflecting pharmacological or hormonal differences in drug metabolism [82].

The complexity of these interactions highlights the importance of an individualized approach to treatment. Monitoring drug levels and antibody formation allows clinicians to distinguish between inadequate exposure, immune responses, or resistance mechanisms, identify the cause of therapeutic failure, and tailor treatment accordingly [76]. In this context, it is necessary to consider the impact of body weight, including the interaction of obesity with the choice of the next, but also the first, biologic drug, given the observed variability in response to treatment associated with obesity.

Evidence from the available literature indicates that even modest weight reduction, achieved through dietary modification, increased physical activity, or pharmacologic agents such as GLP-1 receptor agonists, can improve disease activity and enhance responsiveness to biologic treatment [83,84]. Weight loss may impact multiple levels that represent treatment success, including systemic inflammation, insulin sensitivity, adipokine balance, and endothelial dysfunction [84]. Therefore, in addition to benefits related to affected muscle and bone structures, normalization of body weight may have far-reaching positive effects on overall prognosis.

In summary, obesity is associated with biological therapy outcomes in SpA. Excessive body weight affects pharmacokinetic changes that reduce drug exposure, as well as pharmacodynamic resistance that weakens treatment effectiveness. Together, these mechanisms contribute to shorter drug survival and less favorable long-term outcomes. Since obesity is a modifiable component of SpA, it should be addressed in a targeted manner to improve therapeutic response and prolong drug survival. Simultaneous control of metabolic and overall disease activity provides the best opportunity to optimize therapeutic outcomes and enhance patient quality of life.

## 7. Cardiometabolic and Multisystem Burden

SpA is accompanied by a range of cardiometabolic and psychosocial comorbidities that extend beyond musculoskeletal inflammation [85]. Patients with axSpA experience increased cardiovascular morbidity and mortality, mainly due to systemic inflammation and conventional risk factors such as hypertension, hyperlipidemia, diabetes, and obesity [86]. In the Groningen Leeuwarden axSpA cohort of 461 participants, the prevalence of overweight was 37% and obesity 22%, compared with 43% and 15%, respectively, in age- and sex-matched controls. Patients with axSpA and obesity had higher levels of inflammatory markers and significantly worse disease activity, function, and quality of life compared with overweight and normal-weight patients [12]. Obesity was also identified as an independent predictor of worse clinical outcomes in this population [12]. Obesity is associated with MetS, which is significantly linked to cardiovascular risk assessed by the SCORE equation and to arterial stiffness in the SpA population [87]. Results from a cohort of 295 patients with axSpA showed that the incidence of cardiovascular events was significantly associated with traditional cardiometabolic risk factors (including older age, diabetes, hypertension, and lipid-lowering treatments) and sustained inflammatory activity, as indicated by elevated CRP levels and higher ASDAS-CRP scores over time [88]. Underlying several cardiovascular morbidities in SpA is subclinical atherosclerosis, a well-recognized manifestation of the disease [89].

Non-alcoholic fatty liver disease (NAFLD) is part of the cardiometabolic–renal–liver axis, sharing bidirectional links with central obesity, MetS, type 2 diabetes mellitus, dyslipidemia, hypertension, and chronic kidney disease (CKD). NAFLD has been proposed as an independent risk factor for cardiovascular disease, although this remains a matter of debate [90]. Circulating inflammatory markers, including CRP, IL-6, CCL2, leukocyte counts, and Th1/Th17 lymphocytes, increase in parallel with rising obesity and are key drivers in the pathogenesis of NAFLD. In NAFLD, hepatocyte-derived Fetuin-A activates TLR4 signaling, disrupting insulin receptor signaling and amplifying downstream pro-inflammatory cytokine production, thereby reinforcing a cycle linking inflammation, obesity, and NAFLD [91].

In a single-center observational study of 170 patients with AS, NAFLD was present in 57%. Obesity-related anthropometric parameters (BMI, waist circumference, waist-to-height ratio) were identified as predictors of the presence and severity of hepatic steatosis. Patients with NAFLD had higher CRP, ALT, and triglyceride levels, and lower HDL compared to controls. Waist circumference cutoff values of 100 cm for men and 90 cm for women demonstrated high diagnostic accuracy (AUC 0.84–0.94) for NAFLD screening [92].

Among psychosocial factors, sleep duration is a significant and modifiable risk factor for overweight and obesity [93]. Sleep disturbance was present in nearly 50% of patients with SpA and persisted even 10 years after diagnosis, regardless of treatments or social factors [94]. Sleep disturbances in SpA were strongly associated with depressive symptoms and reduced quality of life in a cohort of 330 SpA participants, with 46.6% exhibiting insomnia symptoms. In axSpA, positive associations were found between insomnia symptoms and HLA-B27 positivity, disease activity as assessed by the BASDAI score, and depressive symptoms. Negative associations were observed between insomnia symptoms and both functional capacity and disease duration [95].

Zhao et al. reported a pooled depression prevalence of 15% using a Hospital Anxiety and Depression Scale threshold of ≥11, with affected patients experiencing higher disease activity and greater functional impairment [96]. Compared with controls, patients with SpA had approximately 80% higher odds of depression. Depression in SpA has a complex bidirectional relationship, in which depression may exacerbate disease activity, while disease severity and certain symptoms can contribute to low mood [97]. 

Peripheral inflammation may affect mood, cognition, and behavior through three main routes. Immune cells travel directly to brain vasculature and tissue via meningeal lymphatics. Vagal signaling transmits cytokine signals to the brain through retrograde axonal transport along the vagus nerve. The humoral pathway involves cytokine production by macrophage-like cells in the circumventricular organs, allowing cytokines to enter the brain by volume diffusion [98].

In the ATLANTIS survey, patients with SpA reported substantial impairment in work ability, with nearly half experiencing disability and significant limitations in professional activity, often resulting in increased absenteeism, presenteeism, and indirect socioeconomic costs due to their disease burden [99].

In conclusion, obesity and SpA together create a self-sustaining inflammatory and metabolic state that worsens cardiometabolic risk, psychosocial burden, and long-term clinical outcomes. Understanding the role of obesity and related lifestyle factors in driving these effects provides an important basis for implementing targeted interventions and lifestyle changes in the comprehensive management of SpA.

## 8. Modifiable Pathways: Weight Loss and Lifestyle Interventions

Obesity may exacerbate the pathology associated with SpA and systemic inflammation through adipokine dysregulation (elevated leptin and resistin promoting Th17 polarization, while reduced adiponectin impairs regulatory T cell function), thus amplifying disease activity and therapeutic resistance [68,100,101,102,103,104]. Increasing evidence positions weight loss as a modifiable pathway to mitigate these effects, with interventions encompassing dietary restriction, physical activity, pharmacotherapy, and bariatric surgery having been associated with reductions in inflammatory biomarkers and clinical endpoints such as BASDAI, DAS28, and MDA achievement [68,100,101,102,103,104]. Nonetheless, while mechanistic plausibility and observational data abound, the scarcity of robust RCTs underscores the imperative for integrated, multidisciplinary care models to bridge evidence gaps and optimize patient outcomes [68,100,101,102,103,104].

Adipose tissue in obesity functions as an endocrine organ, secreting pro-inflammatory mediators that intersect with SpA immunopathogenesis. Visceral fat-derived leptin enhances osteoclastogenesis and synovial inflammation, correlating with BASDAI scores in axSpA cohorts (r = 0.42, *p* < 0.01) [68,100,101,102,103]. Epidemiological studies reveal that patients with SpA and obesity exhibit 1.5–2-fold higher disease activity and poorer biologic response rates, with BMI > 35 kg/m^2^ predicting TNFi failure (OR 2.8, 95% CI 1.4–5.6) [68,100,101,102,103]. This interplay rationalizes targeted weight management, yet confounding factors such as physical inactivity and dietary patterns necessitate holistic approaches [68,100,101,102,103].

Dietary Inflammatory Index (DII) quantifies the inflammatory potential of diets, with higher scores linked to increased low-grade inflammation relevant to SpA outcomes. Anti-inflammatory diets, such as the Mediterranean diet rich in omega-3 fatty acids, fruits, vegetables, and olive oil, or alternatives like the DASH diet emphasizing whole grains and low-fat dairy, can lower DII scores and reduce inflammatory markers like CRP in inflammatory conditions [71,105]. These approaches complement weight loss by targeting obesity-associated inflammation relevant to SpA [71,105]. Caloric restriction via hypocaloric (800–1200 kcal/day) or very low-energy diets (VLED, <800 kcal/day) has been associated with anti-inflammatory effects in SpA. A 2025 pilot RCT (*n* = 40 patients with PsA and obesity) employing six-month VLED reported median 18.6% weight loss (IQR 14.2–22.1%), paralleled by 45% CRP reduction (from 12.4 to 6.8 mg/L, *p* < 0.001), decreased cartilage oligomeric matrix protein (COMP), and 65% MDA attainment versus 20% in controls [106]. The DIETA trial, a 12-week RCT in PsA (*n* = 60), demonstrated a hypocaloric diet plus omega-3 fatty acids reduced swollen joint counts (SJC66) by 2.1 (95% CI −3.4 to −0.8) and ultrasound synovitis scores, independent of weight loss magnitude, implicating direct immunomodulation via resolvin pathways [107]. Mediterranean diet (MedDiet) adherence, rich in polyphenols and monounsaturated fats, further attenuates endothelial dysfunction and cytokine burden in SpA; prospective cohorts show 10% weight reduction over 6 months associates with ASDAS-CRP improvement (Delta −1.2 points) [100,107]. Intermittent fasting variants, though underexplored, may mirror these benefits by enhancing autophagy and gut microbiome diversity, relevant to SpA dysbiosis [100,108,109]. Limitations persist: most studies are small (*n* < 100), short-term (<1 year), and lack SpA-specific endpoints, precluding meta-analytic synthesis [100,108,109].

Furthermore, aerobic and resistance training may help alleviate biomechanical stress and inflammation in overweight SpA. A 2024 multicenter trial (*n* = 120 axSpA) of 12-week multicomponent exercise (150 min/week moderate-intensity) achieved 5.2 kg mean loss, BASDAI reduction (−1.8, *p* = 0.002), and CRP normalization in 52% of participants, surpassing diet-alone arms [104,110]. High-intensity interval training (HIIT) optimizes fat mass reduction while preserving lean mass, countering sarcopenia risks in SpA; meta-regression confirms BMI-dependent MDA gains (beta = 0.31 per 5% loss) [104,110]. These interventions may enhance anti-TNF efficacy by mitigating “adipose resistance,” since patients with obesity who lost ≥ 10% weight showed 2-fold ACR20 responses. Adherence challenges, including enthesitis flare risks, are mitigated by hydrotherapy and patient-tailored protocols, yet long-term RCTs (>2 years) are absent [104,110].

Psychological support may enhance motivation for sustaining lifestyle changes and mitigate stress, which can exacerbate weight gain and inflammation via cortisol pathways. Cognitive-behavioral interventions, for instance, improve self-efficacy and long-term adherence to weight loss in patients with obesity, potentially benefiting SpA disease activity [111,112,113]. Integrating such support can address both behavioral and physiological barriers in modifiable pathways [111,112,113].

Regarding anti-obesity pharmacotherapy, GLP-1 receptor agonists (GLP-1RAs) have been shown to promote substantial weight loss in patients with SpA by enhancing satiety, delaying gastric emptying, and improving metabolic profiles, which is particularly relevant given the high obesity prevalence in this population that exacerbates disease activity and treatment resistance [114,115,116]. In PsA, real-world studies demonstrate clinically meaningful weight reductions averaging 6.43 kg within one year of GLP-1RA initiation, with 60% of patients achieving at least 5% body weight loss; these changes correlate proportionally with improvements in systemic inflammation (e.g., reduced CRP), pain, enthesitis, and cardiometabolic markers like triglycerides and LDL cholesterol [117]. Although direct prospective data in axSpA remain limited, preclinical evidence suggests that GLP-1RAs exert weight-independent anti-inflammatory and chondroprotective effects via immunomodulation of key inflammatory pathways, warranting controlled trials to establish their adjunctive role in SpA management alongside disease-modifying antirheumatic drugs [114,115,116].

Tirzepatide (Mounjaro/Zepbound; maximum dose 15 mg subcutaneously weekly) demonstrates superior weight loss efficacy compared to semaglutide (Wegovy; maximum dose 2.4 mg subcutaneously weekly), achieving approximately 20.2% versus 13.7% body weight reduction at 72 weeks in clinical trials, while liraglutide (Saxenda; maximum dose 3 mg subcutaneously daily) yields more modest outcomes. These GLP-1RAs and dual GLP-1/glucose-dependent insulinotropic polypeptide agonists such as tirzepatide promote satiety, slow gastric emptying, and improve glycemic control, suggesting potential value for patients with SpA and obesity where weight reduction alleviates mechanical joint stress and systemic inflammation. In the SpA setting, these agents support therapeutic targets by facilitating sustained weight loss, potentially enhancing biologic response rates [118,119,120,121].

In addition, GLP-1RAs reduce low-grade chronic inflammation in obesity through direct immunomodulatory mechanisms, including adipokine normalization (e.g., decreased leptin and resistin) and suppression of pro-inflammatory cytokines such as TNF-alpha and IL-6, effects that persist beyond weight loss alone [83,115,122]. In SpA, this attenuation may disrupt the adipose-driven cytokine cascade targeting entheses and joints, improving disease activity scores and structural outcomes alongside direct benefits from reduced adiposity [83,115,122]. Emerging evidence from related rheumatologic conditions, including RA and psoriatic disease, underscores their dual role in mitigating cardiometabolic risk and inflammatory burden, warranting integration into SpA management algorithms [83,115,122].

Bariatric surgery in patients with SpA yields mixed outcomes, with substantial weight loss often correlating to improved disease activity in related rheumatic conditions such as PsA and RA, though direct evidence in axSpA remains limited and occasionally adverse [123,124]. In a retrospective analysis of nine patients with axSpA post-bariatric surgery (primarily Roux-en-Y gastric bypass), mean weight reduction was 49.3 kg, yet SpA symptoms, including inflammatory back pain and high BASDAI and ASDAS-CRP scores, emerged or worsened post-procedure, with structural progression (new syndesmophytes) in over half and one case requiring surgical reversal for refractory disease [125]. Conversely, broader reviews indicate benefits in rheumatic cohorts with obesity, such as remission rates rising from 26% to 74% in patients with RA at 5.8 years follow-up and reduced PsA severity scores (from 6.4 to 4.5), alongside lower MDA thresholds tied to >10% weight loss, suggesting potential adjunctive value despite perioperative risks like complications from immunosuppressants [124]. These findings underscore the need for larger prospective studies to clarify causality, microbiota influences, and long-term efficacy in SpA management. That said, evidence remains observational, lacking sham-controlled trials [123,124,125,126].

Integrated care is imperative for effective weight loss in patients with SpA, as obesity exacerbates disease activity, impairs treatment responses, and heightens cardiometabolic risks, necessitating coordinated multidisciplinary interventions beyond isolated pharmacological or surgical approaches [100,120]. Guidelines from ASAS-EULAR and national societies emphasize holistic management involving rheumatologists, dietitians, physiotherapists, and psychologists to deliver tailored nutritional counseling, supervised exercise, and behavioral strategies, with hypocaloric diets yielding significant inflammation reductions in overweight SpA cohorts [100,126,127]. This patient-centered model addresses the evidence gap in SpA-specific dietary guidance while leveraging synergies, such as ≥5–10% weight loss correlating with lower BASDAI scores and MDA, ultimately enhancing long-term adherence, comorbidity prevention, and quality of life [100,125,126].

In conclusion, evidence gaps persist in weight loss interventions for patients with SpA, particularly regarding causality between adiposity reduction and disease modification, optimal modalities (e.g., diet versus pharmacotherapy), and generalizability across axial versus peripheral subtypes, as most data derive from small, high risk-of-bias studies in PsA rather than prospective axSpA trials [100,127]. The current literature highlights heterogeneous outcomes from hypocaloric diets, with benefits like improved anti-TNF responses and BASDAI scores tied to ≥10% weight loss, yet lacking mechanistic clarity on microbiota or cytokine pathways, compounded by sparse longitudinal data on sustained remission or radiographic progression [100,126]. Future directions include multicenter RCTs evaluating GLP-1RAs, bariatric surgery, and integrated lifestyle programs against standardized endpoints (e.g., ASDAS-CRP, MDA), alongside biomarkers for personalized approaches and cost-effectiveness analyses to inform guidelines [100,126,127].

## 9. Future Directions and Conclusions

The interaction between obesity and SpA represents an important and specific example of how metabolic and immunological pathways intersect to shape chronic inflammation and influence treatment outcomes. Given the growing body of research, obesity is increasingly considered more than a comorbidity. Rather, it may act as an active driver of immune dysregulation that affects disease activity, structural damage, and pharmacological response [71]. The next generation of translational and clinical research should focus on elucidating mechanistic links between adipose-mediated inflammation and therapeutic efficacy, while identifying strategies to personalize treatment for this expanding patient population.

Recent research is changing the way chronic inflammatory conditions are understood. In SpA, metabolic stress appears to reshape the behavior of both immune and stromal cells by altering activation thresholds and cytokine programs [55]. Excess body fat promotes a pro-inflammatory state that can reinforce M1-skewed macrophage activity and Th17-leaning immunity, with downstream relevance for IL-23 and IL-17 signaling at the entheses and synovial membrane. This metabolic inflammation may contribute to a self-sustaining cycle in which systemic cytokines exacerbate insulin resistance, oxidative stress, and mitochondrial dysfunction, thereby further amplifying inflammation and tissue remodeling [18]. Over time, these disruptions may contribute not only to inflammatory activity but also to structural change and abnormal bone growth that are hallmarks of SpA.

This framework supports the rationale for adjunct metabolic strategies in addition to standard disease-modifying therapy. Drugs such as GLP-1RAs and SGLT2i have shown anti-inflammatory and endothelial-protective effects that may enhance the efficacy of biological treatments. GLP-1RAs, in particular, reduce circulating inflammatory mediators, improve insulin sensitivity, and facilitate weight loss [128]. Although such approaches are associated with improved disease control in rheumatic diseases, prospective SpA-specific studies are needed to clarify the utility, timing, and patient selection for these agents. In parallel, non-pharmacological strategies such as tailored nutrition and structured exercise remain essential to restore metabolic balance and support integrated patient care [129].

Targeted treatment through precision medicine forms the basis of SpA management. Advances in pharmacokinetic modeling and body composition assessment could ultimately lead to dose optimization tailored to individual metabolic phenotypes. Quantitative approaches, including fat-free mass, inflammatory biomarkers, and pharmacogenomic data, may enable more accurate prediction of biologic drug exposure and response [130]. Therapeutic drug monitoring models represent a practical next step, with potential to provide feedback that improves drug selection, dosing, and timing of administration, and to support data-driven clinical decision tools.

Multidisciplinary collaboration will be essential to consolidate metabolic and rheumatology care, given the unfavorable cardiovascular risk profile in SpA, particularly in patients with obesity. The higher prevalence of arterial hypertension, dyslipidemia, and NAFLD in these patients further worsens systemic inflammation and contributes to poorer long-term outcomes [73,131]. A coordinated team composed of a rheumatologist, endocrinologist, nutritionist, and physiotherapist can facilitate early metabolic screening, lifestyle intervention, and continuous monitoring. Structured weight loss programs and supervised exercise regimens have already demonstrated reductions in disease activity and improvements in treatment retention, highlighting tangible results relevant to routine clinical practice [132].

Future prospective studies should prioritize resolving the pathophysiological mechanisms underlying the association between obesity and response to therapy in SpA. Randomized clinical trials examining combinations of metabolic and biological therapy could provide high-quality evidence for additive or synergistic effects. Translational studies investigating adipokine signaling, mitochondrial metabolism, and stromal–immune cell communication in enthesis-derived tissues may reveal biomarkers that predict disease course and therapeutic response [56,73]. Biomarker-driven stratification could enable earlier identification of patients who might benefit from intensified or combined therapy and could inform treatment sequencing across biologics and targeted metabolic agents.

In conclusion, linking obesity to therapeutic and multisystem domains of SpA is central to a modern evidence-based medicine approach. Progress in integrating systemic inflammation with excess body weight and metabolic function creates opportunities for innovation across pharmacological, lifestyle, and precision medicine. Despite progress in the last decade, critical gaps remain in optimizing obesity management in SpA. Continued translational and clinical research is needed to improve long-term outcomes, strengthen treatment adherence, and refine therapeutic strategies. Ultimately, integrating metabolic control with immunomodulatory therapy may transform SpA care from disease suppression to holistic, mechanism-driven treatment.

## Figures and Tables

**Table 1:** Semi-quantitative summary of inflammatory mediators reported in SpA patients with and without obesity [18,19,20,21,28,29,30,31,34,35,36,37,40,41,44,45].

SpA with Obesity	SpA Without Obesity
↑ IL23	↑ IL23
↑↑ IL17A/IL17F	↑ IL-17A/IL17F
IL12	IL 12
IL 22	↑ IL 6
↑↑ TNF-α	↑ TNF-α
↑↑↑ IL 6	MCP-1
IL 1β	
IL8	
MCP-1	

Abbreviations: IL—interleukin; MCP—monocyte chemotactic protein; SpA—spondyloarthritis; TNF-α—tumor necrosis factor alpha. ↑—elevated, ↑↑ moderately elevated, ↑↑↑ significantly elevated.

## Data Availability

No new data were created or analyzed in this study.
